# Exploring the Spatial Variation in the Microbiota and Bile Acid Metabolism of the Compound Stomach in Intensively Farmed Yaks

**DOI:** 10.3390/microorganisms12101968

**Published:** 2024-09-28

**Authors:** Shichun He, Zaimei Yuan, Sifan Dai, Zibei Wang, Shusheng Zhao, Bin Zhang, Huaming Mao, Dongwang Wu

**Affiliations:** 1 Yunnan Provincial Key Laboratory of Animal Nutrition and Feed, Faculty of Animal Science and Technology, Yunnan Agricultural University, Kunming 650201, China; heshichun0529@163.com (S.H.); 15987179618@163.com (S.D.); 18487142776@163.com (Z.W.); 18487123523@163.com (S.Z.); 2 Kunming Animal Disease Prevention and Control Center, Kunming 650106, China; yy_yzm2023@163.com; 3 Yunnan Academy of Animal Husbandry and Veterinary Sciences, Kunming 650224, China; binzhang89@163.com

**Keywords:** yak, stomach, intensive farming, 16S rRNA, bile acid metabolism

## Abstract

Yaks are one of the important livestock on the Qinghai–Tibet Plateau, providing abundant dairy and meat products for the local people. The formation of these dairy and meat products mainly relies on the microbiota in their gastrointestinal tract, which digests and metabolizes plant feed. The yak’s gastrointestinal microbiota is closely related to the health and production performance of the host, but the molecular mechanisms of diet-induced effects in intensively farmed yaks remain to be elucidated. In this study, 40 chyme samples were collected from the four stomach chambers of 10 intensively farmed yaks, and the bacterial diversity and bile acid changes in the rumen (SFRM), reticulum (SFRC), omasum (SFOM), and abomasum (SFAM) were systematically analyzed using 16S rRNA sequencing and bile acid metabolism. Our results showed that the gastrointestinal microbiota mainly distributes in the four-chambered stomach, with the highest microbial diversity in the reticulum. There is a highly negative correlation among the microbiota in the four chambers. The dominant bacterial phyla, Bacteroidota and Firmicutes, were identified, with *Rikenellaceae*_RC9_gut_group being the dominant genus, which potentially helps maintain short-chain fatty acid levels in the stomach. In contrast, the microbiome within the four stomach chambers synergistically and selectively altered the content and diversity of bile acid metabolites in response to intensive feeding. The results of this study provide new insights into the microbiota and bile acid metabolism functions in the rumen, reticulum, omasum, and abomasum of yaks. This can help uncover the role of gastrointestinal microbiota in yak growth and metabolic regulation, while also providing references for improving the production efficiency and health of ruminants.

## 1. Introduction

Ruminants have an extremely complex digestive system (rumen, reticulum, omasum, and abomasum), which harbors a diverse range of microorganisms, including bacteria, fungi, archaea, viruses, and protozoa [1,2]. These microorganisms play a crucial role in converting low-quality feed resources into the energy needed by the host, thus enhancing production efficiency [3]. Rumen microorganisms within the digestive tract synergistically break down intricate plant fibers and polysaccharides that are prevalent in feed materials such as straw, silage, hay, and grass. This process produces volatile fatty acids, vitamins, and microbial proteins that ruminants can utilize to meet their nutritional needs. The digestive metabolism and nutrient absorption in ruminants are heavily dependent on the functions and roles of the four stomach chambers, three of which are referred to as the forestomach, composed of the rumen, reticulum, and omasum. The forestomachs house an extensive community of microorganisms that facilitate the anaerobic breakdown of proteins, crude fiber, and various other nutrients [4,5]. Furthermore, a substantial correlation exists between the composition of the microbial communities within the forestomach and the metabolic pathways they influence [6]. The rumen microbial community in ruminants differs from symbiotic microbial communities found in other monogastric animals in terms of population density, diversity, and a more complex network of interactions. The interactions within the rumen microbial community encompass a spectrum of dynamics, including competition, mutualism, predation, parasitism, and cross-species symbiosis. [7,8]. The fermentative metabolic functions of the rumen microbial community lead to the breakdown of large particles in feed into smaller, diffusible molecules. Throughout the fermentation process within the rumen, there is an accumulation of volatile fatty acids, carbon dioxide, and hydrogen, which are byproducts of microbial metabolic activities [9]. Therefore, understanding the crucial role of the rumen microbial community in the physiological metabolism of ruminants is highly significant. The energy produced through rumen digestion is almost entirely responsible for providing nutrition to the host animals [10].

The alpine grasslands of northwestern Yunnan, China, play a crucial ecological role, influencing the entire ecosystem of the northwestern Yunnan Plateau. Ruminant animals living in these high-altitude regions have evolved various adaptive traits to withstand harsh environmental conditions, particularly the cold, low oxygen levels, high altitudes, intense UV radiation, and scarcity of forage in the alpine pastures [11]. Despite facing challenges in improving pasture resources, some herders in the region have adopted intensive breeding practices for domesticated yaks, aiming to enhance the survival conditions of ruminant animals in harsh environments. For plateau ruminants, these challenging conditions affect not only the host’s nutritional metabolism but also the symbiotic gut microbial communities, especially in the rumen and omasum, influencing both their abundance and composition. Several studies have identified tripartite interactions among host–diet–rumen microbial communities [12,13,14]. Therefore, understanding the role of gut microbial communities in alpine ruminants is of significant importance. These microbes co-evolve with their hosts, providing metabolic energy necessary for the survival and growth of the host animals [15]. Research has delineated the seasonal fluctuations in the composition of rumen microbial communities in ruminants and their interplay with shifting dietary regimens, especially in contexts where limited pasture resources pose a challenge to the development of local livestock. This insight is instrumental in facilitating the resilience of these animals in the face of harsh environmental conditions [16]. Key factors driving the survival and development of yaks in high-altitude regions include increased production of volatile fatty acids (VFAs) in the foregut, enhanced microbial fiber degradation capabilities, and reduced methane production. These serve as indicators of efficient energy harvest and nutrient utilization by the microbial community, crucial for sustaining the host during periods of nutritional scarcity. The energy produced by rumen digestion nearly entirely supplies the nutritional needs of the host animal. Therefore, understanding the critical role of the rumen microbial community in physiological metabolism under intensive farming conditions is of significant importance [17]. Therefore, to advance our understanding of how ruminants adapt to high altitudes, it is crucial to analyze the diversity of stomach microbial communities under intensive feeding practices. This analysis helps identify the co-evolutionary adaptations between the host and microbial communities, providing new insights into nutritional management and enhancing our understanding of yak adaptability. Hypothesis: The intensive feeding of yaks leads to significant changes in the microbial diversity and bile acid metabolism in the four stomach chambers, with the expectation that certain bacterial groups (such as Bacteroidota and Firmicutes) become more dominant and that these shifts correlate with alterations in bile acid profiles that influence digestion and nutrient absorption efficiency.

## 2. Materials and Methods

### 2.1. Experimental Animals and Sample Collection

In Shangri-La City, Yunnan Province, China, at Tiancheng Lunzhu Livestock Technology Co., Ltd., a total of 10 healthy 3-year-old male yaks were selected for intensive feeding from August to November 2022. The justifications for using 3-year-old yaks were related to their maturity and the stability of their microbiota at this age, ensuring more consistent results. One time point was chosen to simplify the study and due to resource constraints. The decision to use males exclusively was based on various factors, such as hormonal differences between sexes that influence gastrointestinal microbiota and bile acid metabolism. Using males might help to control these variables and reduce potential confounding factors. During the experimental period, the daily ration consisted of whole-plant ensiled corn and concentrate feed (nutrient composition detailed in Table 1).

All 10 yaks were slaughtered by professional slaughterhouse operators at the end of the experiment. Skilled butchers immobilized the animal’s head for a precise cut. The animals were then ritually slaughtered without stunning, using a sharp knife to swiftly sever the trachea, esophagus, carotid arteries, and jugular veins. The authorized slaughtermen restrained the head to avoid any movement of the animal that could compromise the effectiveness of the cut. After this step, the animals were slaughtered according to Islamic religious ritual (without stunning) using a knife through the structures at the front of the neck—the trachea, esophagus, carotid arteries, and jugular veins—using cutting techniques.

The procedures were approved by the Animal Protection and Utilization Committee of Yunnan Agricultural University, China (protocol #202207030). Professional butchers separated the gastrointestinal tract and collected contents from the rumen (SFRM), reticulum (SFRC), omasum (SFOM), and abomasum (SFAM), with each portion being aliquoted into 10 mL cryotubes (30 mL per stomach content). These samples were rapidly frozen in liquid nitrogen and transported back to the laboratory for subsequent analyses.

### 2.2. UHPLC-MS/MS High-Throughput Target Quantitative Assay of Bile Acids

Metabolites Extraction: In total, 100 µL sample was added to an EP tube, mixed with 400 µL pre-chilled (−40 °C) acetonitrile-methanol (1:1), vortexed, sonicated for 15 min, incubated at −40 °C for 1 h, and centrifuged at 12,000 rpm for 15 min at 4 °C, and the supernatant was collected for UHPLC-MS/MS.

UHPLC-PRM-MS Analysis: UHPLC was performed on a Vanquish system with a Waters ACQUITY UPLC BEH C18 column (Waters, MA, USA), using ammonium acetate and acetonitrile as mobile phases, at 45 °C column and 4 °C autosampler temperatures, with 1 µL injections. An Orbitrap Exploris 120 mass spectrometer (Thermo Fisher Scientific, Waltham, MA, USA) was used for assay development. The standard ion source parameters were as follows: spray voltage at +3500/−3200 V, sheath gas (N_2_) flow rate at 40, auxiliary gas (N_2_) flow rate at 15, sweep gas (N_2_) flow rate at 0, auxiliary gas (N_2_) temperature at 350 °C, and capillary temperature at 320 °C.

We used the UHPLC-PRM-MS/MS method to detect bile acid compounds in samples and analyze 70 targeted bile acid compounds in samples. Analysis was conducted on contents from the rumen, reticulum, omasum, and abomasum, yielding a total detection of 44 bile acid compounds across the samples.

### 2.3. DNA Extraction and Sample Quality Control

DNA extraction was performed using a DNA Kit (TianGen, Beijing, China, Catalog #: DP712), while the CTAB method was used for other sample types. The rumen fluid samples were sent to Yunnan Pulis Biotechnology Co., Ltd. (Kunming, China) for sequencing, and the 16S rRNA genes from the V3-V4 regions were amplified with the barcoded primer of Dao et al. (341F: 5′-CCTAYGGGRBGCASCAG-3′, 806R: 5′-GGACTACNNGGGTATCTAAT-3′) [18]. PCR was conducted with 15 µL of Phusion^®^ High-Fidelity PCR Master Mix (New England Biolabs, Ipswich, MA, USA), 2 µM each of forward and reverse primers, and approximately 10 ng of template DNA. The cycling conditions included an initial denaturation at 98 °C for 1 min, followed by 30 cycles of denaturation at 98 °C for 10 s, annealing at 50 °C for 30 s, and elongation at 72 °C for 30 s, with a final extension at 72 °C for 5 min. PCR products were mixed with an equal volume of 1× loading buffer (containing SYBR Green) and separated on a 2% agarose gel. The bands were purified using a Universal DNA Purification Kit (TianGen, China, Catalog #: DP214), and sequencing libraries were prepared with the NEB Next^®^ Ultra^TM^ II FS DNA PCR-free Library Prep Kit (New England Biolabs, USA, Catalog #: E7430L) according to the manufacturer’s instructions, with indices added. Library quantification was performed using Qubit and real-time PCR, and size distribution was analyzed with a bioanalyzer. The quantified libraries were pooled and sequenced on Illumina platforms, based on the effective library concentration and data requirements.

The raw sequencing data were first processed by concatenation and filtering to obtain clean data. Subsequently, denoising was performed using DADA2 or deblur (defaulting to DADA2) on the clean data to generate Amplicon Sequence Variants (ASVs). These ASVs were then annotated for species information based on their representative sequences, obtaining species-level abundance distributions. Simultaneously, analyses of abundance, alpha diversity, and Venn diagrams were performed on the ASVs to assess species richness and evenness within samples, as well as to identify shared and unique ASVs across different samples or groups.

Multiple sequence alignments of the ASVs were performed, and a phylogenetic tree was constructed. Principal Coordinates Analysis (PCoA) was employed to examine variations in community structures among different samples or groups. The LEfSe method was employed to assess significant differences in species composition and community structure among grouped samples. Additionally, functional predictions of microbial communities in ecological samples were conducted using PICRUSt2 software (https://github.com/picrust/picrust2/wiki).

### 2.4. Statistical Analysis

Spearman’s correlation analysis function in R language 4.3.0 was used to calculate the correlation between abundance data of species at the genus level. After the species correlation coefficient matrix was obtained, the connections with correlation coefficient < 0.6 were removed, the node self-connections were filtered out, and the connections with node abundance less than 0.005% were removed. The connections with *p* < 0.05 were selected as valid connections to draw the network diagram.

## 3. Results

### 3.1. Rumen, Reticulum, Omasum, and Abomasum 16S rRNA Revealed the Diversity and Structure of the Microbiome Associated with an Intensive Feeding System

In this study, a total of 1.46 Gbp of sequencing data were generated, comprising 6,421,320 reads in total (131,047 reads per sample). Within the range of 93,116 to 121,045 sequences per sample, we obtained 5,424,490 high-quality 16S rRNA gene sequences. To mitigate the impact of sequencing depth on microbial community identification, we subsampled each sample’s data to the minimum number of reads (93,116 reads).

**Figure 1:**
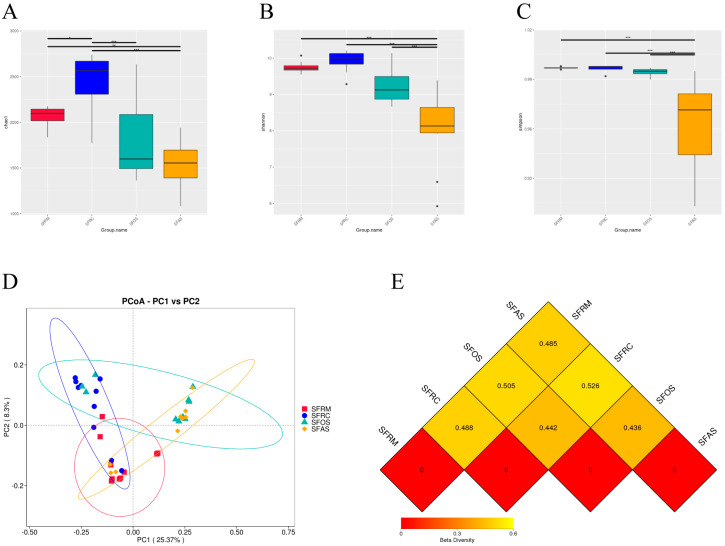
Boxplot of alpha diversity index differences among groups, PCoA plot, and unweighted UniFrac distance matrix heatmap of beta diversity indices. (**A**) Chao1, (**B**) Shannon, (**C**) Simpson, (**D**) PCoA plot, (**E**) unweighted UniFrac distance matrix heatmap. SFRM (rumen), SFRC (reticulum), SFOS (omasum), and SFAS (abomasum). * indicates that the *p* < 0.05. ** indicates that the *p* < 0.01. *** indicates that the *p* < 0.001.

Comparative analysis of diversity indices in intensively fed yaks indicated that the reticulum’s Chao1 diversity index was significantly higher than that of the rumen, omasum, and abomasum (*p* < 0.05) (Figure 1A). The abomasum’s Shannon and Simpson indices were significantly lower than those of the rumen, reticulum, and omasum (*p* < 0.05) (Figure 1B,C).

Principal Coordinates Analysis (PCoA) based on Bray–Curtis dissimilarities revealed distinct clustering of samples corresponding to the rumen, reticulum, omasum, and abomasum, indicating differences in bacterial composition among the four groups (Figure 1D). The SFAS-SFRC dissimilarity coefficient of 0.526 was the highest among pairwise comparisons of other stomachs, suggesting greater microbial diversity between these two stomachs. In contrast, the SFAS-SFOS dissimilarity coefficient of 0.436 was the smallest, indicating lower microbial diversity between these two stomachs (Figure 1E).

### 3.2. Compositional Profiles of the Rumen, Reticulum, Omasum, and Abomasum Bacteria Altered by Intensive Feeding System

**Figure 2:**
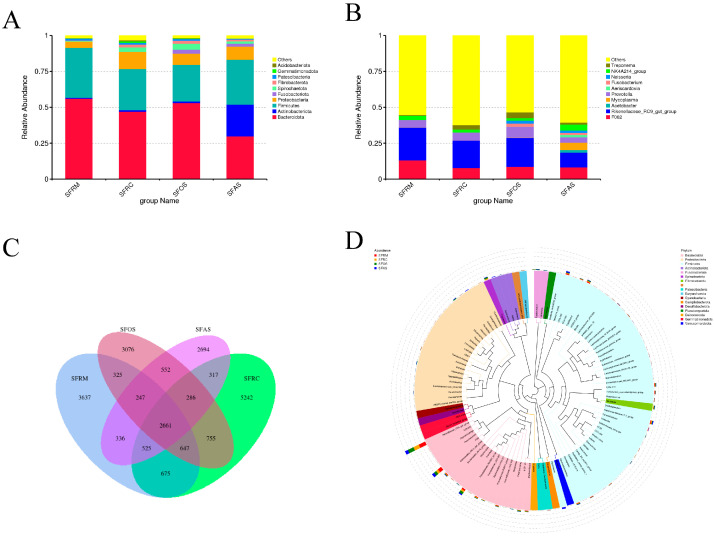
(**A**) Bar chart of the top 10 phylum-level relative abundances, (**B**) bar chart of the top 10 genus-level relative abundances, and (**C**) Venn diagram where each circle represents a sample or group. The numbers in the overlapping regions denote the quantity of shared feature sequences between the samples or groups. Numbers outside of the overlapping areas indicate the number of unique feature sequences specific to each sample or group. (**D**) Genus-level phylogenetic tree: The tree was constructed using representative sequences of genera, with branches and sectors colored to indicate their corresponding phyla. Stacked bar charts positioned outside the outer rim of the sectors illustrate the genus abundance distribution across different samples. The legend on the left provides sample information, while the legend on the right shows phylum-level classification corresponding to the genus-level species. SFRM (rumen), SFRC (reticulum), SFOS (omasum), and SFAS (abomasum).

In the rumen, reticulum, omasum, and abomasum, the dominant phyla were Bacteroidota (56.07%, 47.02%, 53.02%, 29.81%) and Firmicutes (34.86%, 28.63%, 25.53%, 31.29%), with the rumen exhibiting the highest abundance of Bacteroidota and Firmicutes compared to the other three stomachs (Figure 2A). At the genus level, the dominant taxon across all four stomachs was *Rikenellaceae*_RC9_gut_group (22.76%, 19.13%, 20.07%, 10.32%) (Figure 2B).

Based on Venn diagrams, a total of 21,957 feature sequences were detected across the four stomachs, with 2661 sequences shared among them. The rumen had 3637 unique feature sequences, the reticulum had 5242, the omasum had 2694, and the abomasum had 3076 unique feature sequences (Figure 2C).

To further investigate evolutionary relationships at the genus level, representative sequences from the top 100 genera were aligned to construct a phylogenetic tree. Dominant genera were found clustered within three main branches: Bacteroidota, Firmicutes, and Proteobacteria (Figure 2D).

### 3.3. Comparative Analysis of Microbial Differences among the Four Stomach Compartments

**Figure 3:**
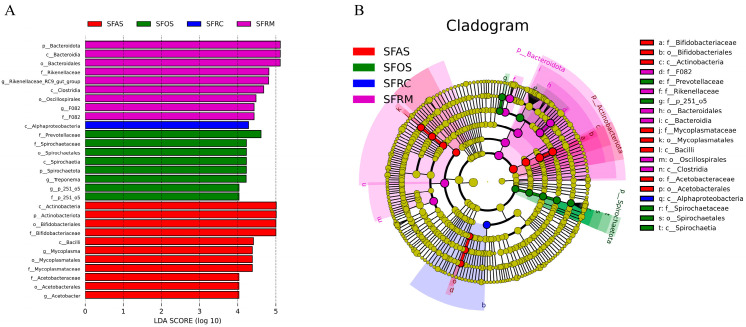
(**A**) LDA (Linear Discriminant Analysis) score distribution bar chart. The “LDA score distribution bar chart” shows species with LDA scores greater than a set value (typically 4), indicating statistically significant biomarkers between groups. (**B**) Evolutionary branching diagram, the “Evolutionary Branching Diagram” features concentric circles radiating outward to depict taxonomic levels from phylum to genus (or species). Each small circle within these levels represents a taxon, with its diameter proportional to its relative abundance. Species with no significant differences are uniformly colored yellow. Biomarker species linked to specific groups are color-coded: red nodes highlight taxa important in the red group, while green nodes denote taxa crucial to the green group. SFRM (rumen), SFRC (reticulum), SFOS (omasum), and SFAS (abomasum).

Using the Lefse analysis method to analyze the microbial community data of yaks’ four stomach compartments under intensive feeding, we identified 29 microbial biomarkers with statistically significant differences (selection criteria: LDA score > 4.0). Among these, the rumen and abomasum exhibited the highest number of differential microbes, with 9 and 11 species, respectively, while the reticulum had the fewest differential microbes, consisting only of Alphaproteobacteria (Figure 3A). Actinobacteria were differential microbes in rumen and abomasum (Figure 3B). These distinct species may explain how intensive feeding alters the abundance of dominant differential microbes in the four stomach compartments of yaks.

### 3.4. Network Correlation Analysis of Bacteria in the Four Stomach Compartments

**Figure 4:**
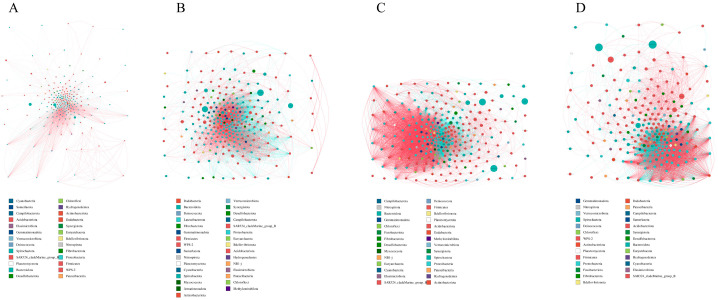
Network diagram: Different nodes in the diagram represent various genera, with node size reflecting the average relative abundance of each genus. Nodes within the same phylum are colored identically (as indicated in the legend). The thickness of the edges between nodes is proportional to the absolute value of the species interaction correlation coefficient, while edge colors indicate whether the correlation is positive or negative (red for positive, blue for negative). (**A**) Rumen, (**B**) reticulum, (**C**) omasum, (**D**) abomasum. SFRM (rumen), SFRC (reticulum), SFOS (omasum), and SFAS (abomasum).

To reveal the relationships among the SFRM, SFRC, SFOS, and SFAS microbial communities in intensively fed yaks, Spearman’s correlation coefficients were computed for all samples at the genus level, resulting in a species correlation coefficient matrix. It was found that the omasum (SFOS) exhibited a more complex bacterial correlation module compared to other stomach compartments (Figure 4). The rumen showed lower bacterial correlations and abundances compared to the other three stomachs (Figure 4A). Bacteria across all four stomachs predominantly exhibited negative correlations at the genus level (Figure 4B). SFOS bacteria displayed higher correlation coefficients and abundances (Figure 4C). SFAS microbial communities exhibited two distinct correlation clusters (Figure 4D). Overall, there was a noticeable increasing trend in both abundance and correlation strength among the SFRM, SFRC, SFOS, and SFAS microbial communities.

### 3.5. Bile Acid Analysis of Bacteria in the Four Stomach Compartments

**Figure 5:**
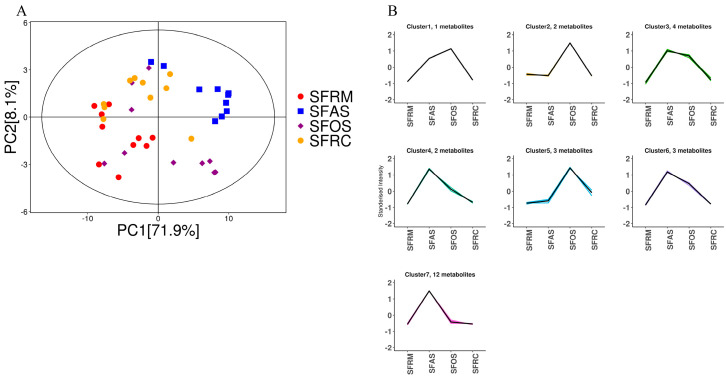
(**A**) Score scatter plot for PCA model TOTAL. (**B**) K-Means analysis for all groups. SFRM (rumen), SFRC (reticulum), SFOS (omasum), and SFAS (abomasum).

Through the quantitative analysis of bile acids in the rumen, reticulum, omasum, and abomasum, the PCA score plot results showed that all samples were within the 95% confidence interval (Figure 5A). The PCA results clearly distinguished the four stomach compartments, with rumen samples being more clustered and omasum samples more scattered. To study the relative changes in metabolite content across different groups, all identified metabolites were normalized by z-score and subjected to K-means clustering analysis. The omasum exhibited higher metabolite content and numbers in Clusters 3, 4, 6, and 7, with 4, 2, 3, and 12 metabolites, respectively (Figure 5B). The abomasum had one, two, and three metabolites in Clusters 1, 2, and 5, respectively.

**Figure 6:**
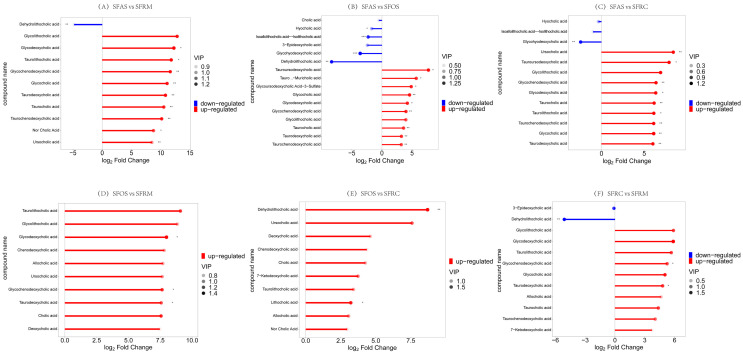
(**A**) Matchstick analysis for group SFAS vs. SFRM. (**B**) Matchstick analysis for group SFAS vs. SFOS. (**C**) Matchstick analysis for group SFAS vs. SFRC. (**D**) Matchstick analysis for group SFOS vs. SFRM. (**E**) Matchstick analysis for group SFOS vs. SFRC. (**F**) Matchstick analysis for group SFRC vs. SFRM. SFRM (Rumen), SFRC (Reticulum), SFOS (Omasum), and SFAS (Abomasum). * indicates that the *p* < 0.05. ** indicates that the *p* < 0.01. *** indicates that the *p* < 0.001.

In the matchstick analysis comparing group SFAS vs. SFRM, nine metabolites showed upregulation, with eight significantly upregulated (*p* < 0.05) and one significantly downregulated (Dehydrolithocholic acid, *p* < 0.01) (Figure 6A). For group SFAS vs. SFOS, 10 metabolites were significantly upregulated (*p* < 0.05), while 6 metabolites were downregulated, with 4 significantly so (*p* < 0.05) (Figure 6B). Comparing group SFAS vs. SFRC, 10 metabolites were significantly upregulated (*p* < 0.05) and 3 metabolites were downregulated (Figure 6C). Matchstick analyses between SFOS vs. SFRM, SFOS vs. SFRC, and SFRC vs. SFRM showed the significant upregulation of 10 metabolites in each comparison (Figure 6D–F).

## 4. Discussion

The gastrointestinal microbiota is a key component of the digestive system in ruminants, significantly influencing the host’s digestion, nutrient absorption, and overall health [3,19]. In recent years, with the transformation of livestock production models, intensive farming systems have played an important role in improving livestock growth performance and feed utilization efficiency [20]. However, the impact of intensive farming systems on the spatial dynamics of the stomach microbiota in yaks has not been fully elucidated. This study explores the effects of intensive farming systems on the stomach microbiota of yaks through deep sequencing technology and bile acid metabolite analysis.

The impact of intensive farming systems on the regional microbiota of the yak stomach is a complex process involving the combined effects of multiple factors. The rumen, reticulum, omasum, and abomasum exhibit certain differences among various ruminants [4,21,22]. The relative abundance of dominant bacterial phyla also changed, with Bacteroidota and Firmicutes being the main dominant phyla, consistent with previous research findings [23,24]. The dominant bacterial genus found in the four stomach compartments was the *Rikenellaceae*_RC9_gut_group. This butyrate-producing bacterium plays an important role in maintaining the levels of short-chain fatty acids (SCFAs) in the gut [25]. At the same time, intensive farming affects the network structure and function of the gastrointestinal microbiota in ruminants [26]. This study indicates that intensive farming systems significantly alter the composition and diversity of the stomach microbiota in yaks. The alpha diversity index of the three stomach compartments, excluding the rumen, showed a gradient decrease: the reticulum had a higher diversity index, while the abomasum had a lower diversity index. This may be related to the different microbial ecological environments in various parts of the stomach under intensive farming systems. Additionally, these findings suggest that intensive farming systems may affect the diversity and composition structure of the stomach microbiota in yaks.

In terms of microbial composition, this study also identified several statistically significant microbial biomarkers, with the most notable differences observed in the rumen and abomasum. In the rumen of yaks grazed at different ages, the key core microorganism belonged to the phylum Firmicutes [27]. Studies indicate that members of Alphaproteobacteria may be associated with functional variations in gut microbiota [28]. In ruminant gastrointestinal tracts, Alphaproteobacteria have evolved specific metabolic or survival capabilities [29]. In this study, Alphaproteobacteria were identified as key differential microbes in the reticulum. These biomarkers may serve as important indicators of how intensive farming systems impact the stomach microbiota of yaks, warranting further investigation. Additionally, microbial network correlation analysis revealed complex relationships among microbiota in different stomach compartments, providing crucial clues for a deeper understanding of the mechanisms through which intensive farming systems affect the stomach microbiota of yaks.

Due to the interaction between gastrointestinal bioactive substances and microbiota [30], microbial-mediated bile acid transformation in the stomach has been shown to be crucial for maintaining intestinal and host health [31,32]. Diet is known to be an important factor influencing the composition of the gut microbiota and the characteristics of host bile acids [33]. The host–microbiota interaction influences bile acid metabolism, thereby affecting the health of the host individual [34,35]. Intensive farming systems have a certain impact on the bile acid metabolism of the stomach microbiota in yaks. This study, through bile acid metabolism analysis, suggests that intensive farming systems may alter both the quantity and types of bile acids metabolized by the stomach microbiota in yaks. Specifically, the abomasum showed higher levels of certain bile acid metabolites, while samples from the omasum exhibited more variability in metabolites. These findings indicate that intensive farming systems could affect the metabolic functions of the stomach microbiota in yaks, thereby influencing the host’s nutrient absorption and health status.

This hypothesis aligns with the observations that the microbiota distribution, composition, and bile acid metabolism vary across the different stomach chambers and are influenced by the intensive feeding regimen. It sets the stage for testing the specific ways in which these microbiota and metabolite changes impact the yak’s digestive health and efficiency.

## 5. Conclusions

This study explores the effects of intensive farming systems on the stomach microbiota and bile acid metabolism in yaks, highlighting potential improvements in growth performance and feed efficiency. These benefits may reduce the pressure on grassland resources, contributing to the preservation of the grassland ecosystem. However, the absence of a control group limits the ability to make definitive claims about the impact of intensive farming. Future research should include comparisons with pasture-fed yaks to better understand how these systems affect the composition and function of the yak’s stomach microbiota and the broader ecological implications.

## Figures and Tables

**Table 1:** Chemical composition of diet and its constituents (% DM basis).

Item	DM	CP	EE	Ash	NDF	ADF	ADL
Whole-plant ensiled corn	67.95	9.27	1.17	12.98	61.32	37.52	2.72
Concentrate	89.64	12.29	2.44	7.90	41.41	10.84	5.24
TMR	55.05	10.48	1.68	10.97	53.21	26.81	2.98

Note: DM: Dry Matter. CP: Crude Protein. EE: Ether Extract. Ash: Crude Ash. NDF: Neutral Detergent Fiber. ADF: Acid Detergent Fiber. ADL: Acid Detergent Lignin.

## Data Availability

The datasets presented in this study can be found in online repositories. Bacterial sequences from this project were deposited in the National Center for Biotechnology Information (NCBI) Short Read Archive (SRA) under the BioProject number PRJNA1036001.
